# Urethral calculi with a urethral fistula: a case report and review of the literature

**DOI:** 10.1186/s13104-017-2798-z

**Published:** 2017-09-06

**Authors:** Mingqiang Zeng, Fanchang Zeng, Zhao Wang, Ruizhi Xue, Liang Huang, Xuyu Xiang, Zhi Chen, Zhengyan Tang

**Affiliations:** 1 Department of Urology, Hunan Provincial People’s Hospital, The First Affiliated Hospital of Hunan Normal University, Changsha, China; 2 Department of Urology, Xiangya Hospital, Central South University, 87 Xiangya Road, Changsha, 410008 Hunan China; 3 Department of Urology, Hainan General Hospital, Haikou, China

**Keywords:** Urethral calculus, Urethral fistula, Urinary tract infections, Suprapubic catheter

## Abstract

**Background:**

To explore and summarize the reasons why urethral calculi cause a urethral fistula.

**Case presentation:**

We retrospectively studied 1 patient in Xiangya hospital and all relevant literature published in English between 1989 and 2015. The patients (including those reported in the literature) were characterized by age, origin, location of calculus, size of calculus, fistulous track, and etiological factors. Most of urethral calculi associated with a urethral fistula were native generated. Urethral calculi can be formed in various locations of the urethra, and the size of the calculus ranged from small (multiple) calculi to giant stones. The fistula external orifice located at the root of the penis was relatively common, and there were various etiological factors, such as urethral strictures, urethral trauma induced by long-term catheterization, lumbar fractures, and congenital anomaly factors. They were managed by the excision of the fistulous tract, retrieval of the urethral stones, and/or debridement and pus drainage operations.

**Conclusion:**

Some elements, such as trauma, recurrent urinary tract infections, abscess formation induced by long-term catheterization, and urethral calculus, may be the risk factors for a urethral fistula.

## Background

Calculi in the urethra are uncommon, representing only 1–2% of all calculi in the genito-urinary tract [1], and urethral calculi causing urethrocutaneous fistula are extremely rare. Only a few cases of urethral calculi causing a urethral fistula have been reported in the literature [2–6]. Here, we present a more complicated case of urethral steinstrasse causing urethrocutaneous fistula with a long-term suprapubic catheter (SPC). Through this work, we hope to gain clinicians’ and patients’ attention concerning urethral calculi with a urethral fistula, a problem which has, so far, been under-reported.

## Case presentation

**Fig. 1:**
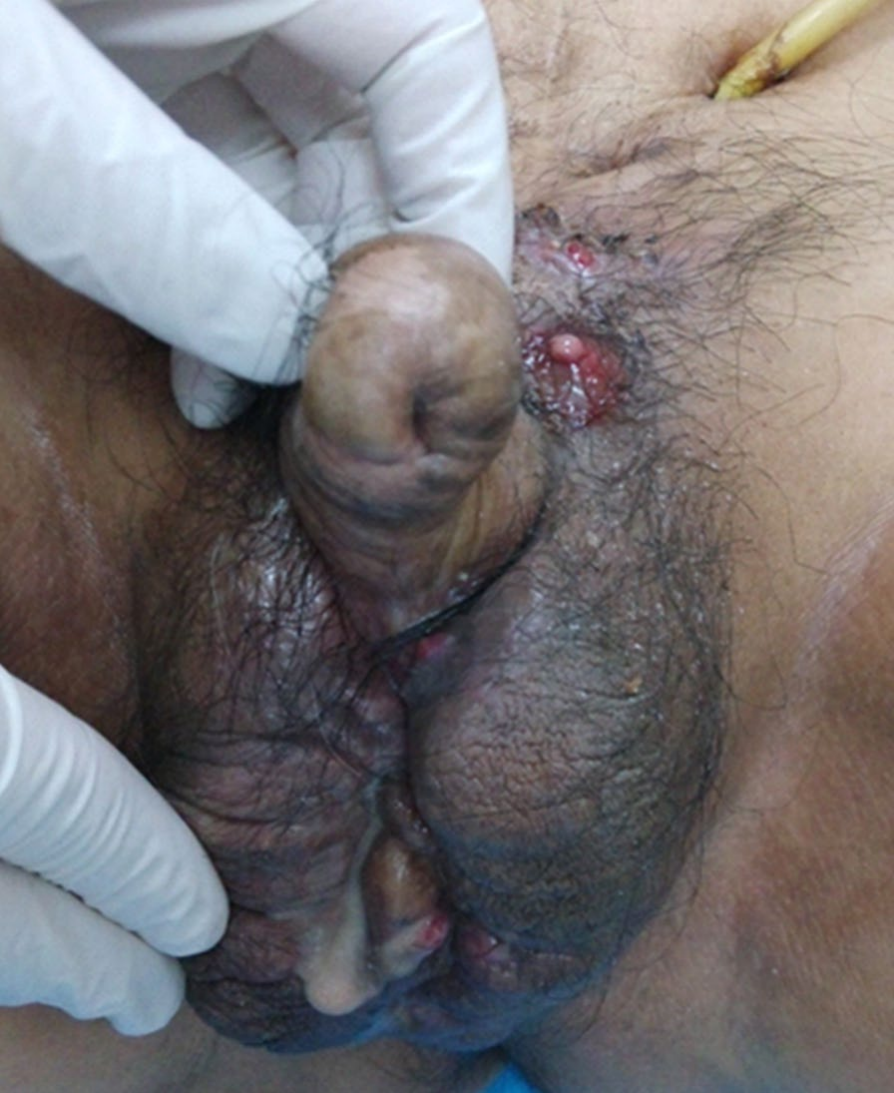
Urethrocutaneous fistula at the ventral side of the penis with a purulent fluid outflow through the fistula, and a long-term suprapubic catheter

**Fig. 2:**
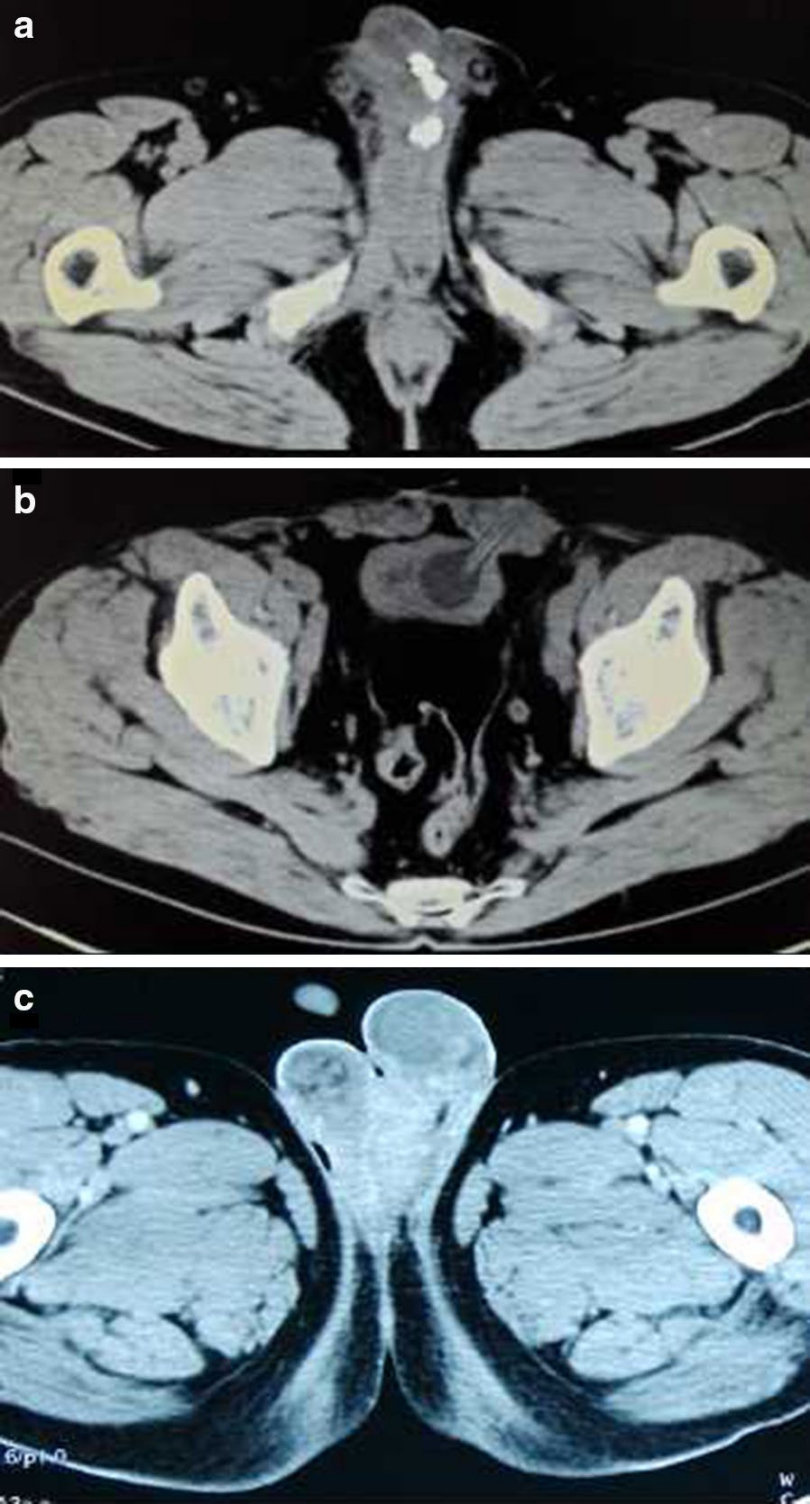
**a** Computed tomography scan of the soft tissue in the penis showing multiple calculi in the distal urethra. **b** Computed tomography scan of the pelvis showing a thickened urinary bladder and Foley’ s catheter through the abdominal wall. **c** Computed tomography scan of the scrotum showing a purulent infection and its spread in the perineal fascia space

A 60-year-old Chinese man experienced pain and swelling of the penile-scrotal area and had a history of the passage of purulent fluid passing through a penile ventral fistula of 3 months’ duration (Fig. 1). He had history of a neurogenic bladder and the prolonged use of the SPC after the suprapubic catheter insertion. The dysfunctional bladder was accompanied with repeated unconscious urine outflow and occasional purulent secretion discharge from the urethra, which eventually progressed to urethral atresia (Fig. 1). The patient did not replace the SPC on schedule and also failed to accept treatment in a timely manner in accordance with his doctor’s advice before the urethrocutaneous fistula formed and the disease progressed. A rectal examination revealed no purulent secretions or rectal fistula formation. Computed tomography (CT) of the soft tissue in the penis revealed multiple calculi in the region of the distal penile area (Fig. 2a) and the suppurative infection of the fascia space in the perineal region (Fig. 2b) accompanied with a thickened urinary bladder (Fig. 2c). After replacing the suprapubic catheter, the patient underwent a debridement and pus drainage operation with the excision of the fistulous tract and retrieval of the urethral stones. Intraoperatively, dense scar tissue of the penile urethra was found, with the complete obliteration in the anterior urethra. The patient was administered intravenous fluids and antibiotics in the perioperative period. After recovery, he returned to the hospital monthly to replace the SPC. The patient’s condition was followed for 6 months, during which time the SPC blockage, severe urinary tract infections (UTIs), urinary calculi, and renal damage did not recur.

## Literature review

We searched PubMed (http://www.ncbi.nlm.nih.gov/pubmed) for articles published in English between 1989 and December 2015 with the terms “urethral calculus”, “urethral steinstrasse”, “fistula”, “urethral fistula”, “urethrocutaneous fistula”, or “recto-urethral fistula”. There were 5 reports included in our study (Table 1). In these studies, all the urethral calculi were native generated (they were formed de novo in the urethra) and formed in different locations of the urethra (in the proximal penile, bulbar, and posterior urethra regions), the size of the calculi ranged from small (the size of a grain of sand) to massive (stones ~6 × 5 cm), and it was common to find the fistula external orifice at the root of the penis. There were also various etiological factors, such as the urethral stricture, urethral trauma induced by long-term catheterization, lumbar fractures, and congenital anomaly factors. Some cases exhibited anatomic abnormalities, such as having a stone in the large diverticulum. Initially, these cases were managed with suprapubic cystostomy and intravenous antibiotics. Later, the patients underwent external urethrotomy and stone retrieval.

**Table 1:** Urethral calculus associated with urethral fistula

No.	Case load	Age	Ethnicity	Native/migratory^a^	Location of calculus	Size of calculus (cm)	Fistulous track	Etiological factors
1	1	38-year-old	Indian	Native	Penobulbar junction [2]	6 × 5	Near the penoscrotal junction	Stricture of penile urethra distal^b^
2	1	41-year-old	Osmanli	Native	At the prostatic urethra [3]	5.9 × 3.2 × 2.8	Below the radix of penis	Urethral trauma as a result of long-standing urethral catheter drainage
3	1	30-year-old	English	Native	Recto-urethral fistula [4]	5 × 3.5	The membranous urethra	Born with abnormalities
4	1	40-year-old	Indian	Native	In proximal penile, bulbar, and posterior urethra [5]	Multiple calculi	At the root of the penis	Urethral stricture
5	1	37-year-old	Japanese	Native	Prostatic urethral [6]	4.2 × 3.4 × 2.1	Urethrocutaneous fistula in the perineal region	A lumbar vertebral fracture

^a^Native: those formed de novo in the urethra. Migratory: those formed in the bladder or kidney with secondary descent

^b^Existed anatomic abnormality, the stone in large diverticulum

## Discussion

Urethral calculi accounted for only 1–2% of the urinary tract stones. Urethral calculi that cause urethral fistula are extremely rare [1], but they still cause serious discomfort in many patients with the disease. In order to obtain a deep understanding of the disease, we analyzed the origin of the urethral calculi and summarized the causes of the urethral calculi that result in a urethral fistula.

### The origin and management of the urethral calculus

Urethral calculi are either formed in the native urethra or migrate from the upper urinary tract [7]. Primary native calculi (those formed de novo in the urethra) are usually small and come in multiples, and secondary migratory calculi (those formed in the upper urinary tract with secondary downward descent) are usually large [8]. We found that the size of the primary native urethral calculi ranged from 2 to 3 mm to 6 cm, larger calculi usually occur in the posterior urethra and vesico-prostatic urethra, and a small calculus is commonly found in the anterior urethra. Primary urethral stones are generally composed of magnesium ammonium phosphate (struvite) [8]. This finding is consistent with our investigation of the stone components. The stones are formed in the urethra either behind some stricture or within a poorly drained communicating cavity, with an obstruction, stagnation, infection, and/or inflammation acting as the predisposing factor [9]. The above predisposing factors exactly explain the formation of various urethral stones in the studies. Secondary or migratory stones are usually composed of calcium oxalate or citrate [3], and they are very common. Migratory stones are most often encountered in association with urethral stricture disease or other forms of urethral obstruction [10].

The main symptoms are acute urinary retention, frequency, a burning sensation in the urethra during urination, a burning sensation in the perineum and/or rectum, or a stinging in the anus. Other less common symptoms included haematuria, dribbling or incontinence, interruption of the urinary stream, and a history of having passed a stone. Management of urethral calculi varied according to the site, size, and associated urethral disease. Retrograde manipulation into the urinary bladder followed by litholapaxy or lithotripsy is a suitable procedure for small urethral calculi. Anterior urethral calculi can be removed with surface anesthesia, endoscopic removal, or ventral meatotomy [11].

### Etiology and treatment of the urethral fistula

Urethral fistulas may be acquired or congenital. Acquired urethral fistulas may be neoplastic, traumatic, or caused by a urethral foreign body or infection [12–15]. Urethral fistulas have been found after a straddle injury [16] and blunt penile trauma [17]. They can also occur as a complication of penile surgery. Urethral fistulas have been described after circumcision [18] and operations for priapism [19]. In this study, we found that the majority of the urethral fistulas were found were secondary to urethral calculi complicated by trauma and infection. They may have occurred as a result of a penile-scrotum abscess. The patients with primary urethral stones were usually asymptomatic or had chronic voiding problems. An important cause of a urethral fistula may be related to delayed treatment. A congenital urethral fistula represents either an embryonic urethral blowout behind a distal congenital obstruction or segmental embryonic arrest so that the mesoderm fails to encircle the developing groove at the site of the fistula [12]. Congenital urethral fistulas may be associated with anorectal atretic malformations, which is consistent with the No. 3 case in Table 1. The fistula of the membranous urethra extended into an abscess cavity which contained the calculus and communicated with the rectum. In the most recent studies, all patients underwent the excision of the fistulous tract and retrieval of the urethral stones [2–6], while only some of the patients with a penile-scrotum abscess underwent a debridement and pus drainage operation. It was necessary to administer antibiotics to the patients during the perioperative period.

### Urethral steinstrasse causing urethrocutaneous fistula with a long-term suprapubic catheter

The SPC is a popular method for managing long-term bladder drainage in voiding dysfunction [20]. Although a SPC has a high success rate, there can be several complications from its use, including UTIs, bladder stones, upper tract calculi, renal scarring, vesicoureteral reflux, urethral incontinence, and even bladder cancer [21, 22]. Urethral steinstrasse was likely caused by repeated UTIs with a long-term SPC. These stones formed in the bladder with a secondary migration to the urethra that was complicated with trauma and infection, which ultimately resulted in the urethral fistula. There were 2 concurrent fistulas in the patient involved in this study, the penile fistula caused by the calculi injuries and the scrotal fistula caused by a serious fascia space infection.

## Conclusions

This study identified some predisposing factors of urethral calculus with a urethral fistula. Patients suffering from this problem can be treated with the excision of the fistulous tract, retrieval of the urethral stones, and/or a debridement and pus drainage operation. The complications seriously affect the quality of life and even cause more serious consequences, so clinicians and patients should be aware of them.

## Abbreviations

SPC: suprapubic catheter
CT: computed tomography
UTIs: urinary tract infections
